# Cardiovascular Responses, Perceived Exertion and Technical Actions During Small-Sided Recreational Soccer: Effects of Pitch Size and Number of Players

**DOI:** 10.2478/hukin-2013-0049

**Published:** 2013-10-08

**Authors:** Alper Aslan

**Affiliations:** 1 Mustafa Kemal University, School of Physical Education and Sports, Antakya, TURKEY.

**Keywords:** Exercise intensity, heart rate, rate of perceived exertion, notation analysis, recreational soccer

## Abstract

The aim of this study was to determine the cardiovascular, perceived exertion and technical effects of altering pitch size and number of players in recreational soccer match-play. The further aim was to evaluate to what extent exercise intensity during various game formats corresponds to the recommended intensity level for cardiovascular fitness improvement. Ten male recreational players aged 31.7±7.6 years (mean ± SD) completed four variations of small-sided games (except for goalkeepers, 5-a-side and 7-a-side on small and large pitches) during which heart rate, perceived exertion and technical actions were evaluated. Two-way analysis of variance on repeated measures was applied to collected data. The results indicated that an average workload expressed as heart rate and percentage of heart rate reserve during 5-a-side games was higher than for 7-a-side games. The rate of perceived exertion values were moderate and similar for all formats of games. The players performed more dribbling and successful passes, but fewer unsuccessful passes during 5-a-side games. Furthermore, the number of ball possessions and unsuccessful passes was higher on a small pitch than on a large one. Consequently, the current findings suggest that, independent of pitch size, the cardiovascular demands imposed on participants increase when the game is played with fewer players. However, all formats of recreational soccer can be used as an effective activity to promote cardiovascular fitness. Finally, participants may have more chance to perform basic technical actions during 5-a-side games on small and large pitches.

## Introduction

Recreational soccer usually takes the form of small-sided games with fewer than eleven players per side, played on a relatively small pitch, mostly on artificial grass, sand carpet, inlaid floor and even on asphalt. It is estimated that, globally, several hundred million people of all ages and genders participate recreationally in soccer. Considering the large number of participants, researchers are becoming increasingly interested in the health and fitness related benefits of recreational soccer (Krustrup et al., 2009; Randers et al., 2010).

Heart rate (HR) monitoring is a widespread indirect method to evaluate physiological stress imposed on players during recreational, amateur and professional games (Ali and Farrally, 1991; Bangsbo, 1994; Casamichana and Castellano, 2010; Kelly and Drust, 2009; Randers et al., 2010). Observations of diverse populations have demonstrated that average cardiovascular load during recreational soccer exceeded 80% of maximum HR (HRmax) (Bertolaccini et al., 2010; Bondarev, 2011; Randers et al., 2010; Randers et al., 2011). This relative workload is similar to that observed for amateur and professional soccer match-play (Bangsbo, 1994; Aslan et al., 2011). Institutions have recommended that the intensity of the physical activity should be between 60 to 85% of HRmax to improve cardiovascular fitness (ACSM, 2000). However, HR values obtained in previous studies (Bertolaccini et al., 2010; Bondarev, 2011; Randers et al., 2010) demonstrated that the intensity in certain periods of a recreational game is higher than a recommended intensity level for cardiovascular safety. Due to the intermittent nature of soccer, it is not possible to constrain the intensity of activities within recommended guidelines throughout the game. Recent studies of professional and amateur soccer players showed that some modifications in the game format influenced the metabolic demands of the games (Bondarev, 2011; Casamichana and Castellano, 2010; Randers et al., 2010; Rampinini et al., 2007; Owen et al., 2004). However, the results of these studies are not consistent. It is of importance to note that in order to isolate the effects of the number of players on exercise intensity, the shape and size of the playing area should be kept unchanged (Rampinini et al., 2007). However, in majority of reports, the playing area was changed with variations in the number of players involved (Little and Williams, 2006; Randers et al., 2010; Casamichana and Castellano, 2010). Furthermore, game duration was also generally increased as the number of players engaged in the activity increased (Little and Williams, 2006; Randers et al., 2010). Nonetheless, there is still a discussion about how cardiac responses are affected by variation of pitch size and the number of players in diverse groups of participants.

Rate of perceived exertion (RPE) is an alternative method to assess the internal load imposed on players during training and match-play. A strong relationship has been established between RPE and HR (Borg, 1982). Moreover, it is suggested that RPE is more sensitive to accumulated fatigue than HR during prolonged exercise (Martin and Andersen, 2000), and may be a more reliable measure of exercise intensity during intermittent activities such as soccer training and match-play (Coutts et al., 2009). Therefore, measuring RPE values may also contribute to better understanding of the internal load imposed on recreational players and accumulated fatigue towards the end of a game.

On the other hand, from the players’ perspective, participation in an activity is not only related to perceived health benefits. It was emphasized that intrinsic motivation entails participation in an activity out of pleasure and enjoyment (Vallerand et al., 2007). It is also well established that enjoyment is a key predictor of commitment and adherence to physical activity (Toh et al., 2011). To this extent, technical actions frequently performed during games may be accepted as an indicative of player enjoyment and satisfactions associated with games. In previous studies conducted on young and professional players, it has been found that small-sided games offer many positive challenges that satisfy player enjoyment, such as the opportunity to repeatedly touch the ball and experience basic tactical challenges (Casamichana and Castellano, 2010; Jones and Drust, 2007; Katis and Kellis, 2009; Kelly and Drust, 2009). However, there is little data regarding technical actions performed during recreational games.

The aim of the present study was therefore to determine the cardiovascular, perceived exertion and technical effects of altering both pitch size and the number of players in recreational soccer match-play. The further aim was to evaluate to what extent exercise intensity during various game formats corresponds to the recommended intensity level for cardiovascular fitness improvement.

## Material and Methods

### Participants

Ten male recreationally active university staff volunteered for this study. At the time of the study, the participants were playing small sided soccer once or twice a week regularly for three to six months, each game lasting from 40 to 60 minutes. However, they continued their sedentary lifestyle during the rest of the week. All subjects fulfilled the following inclusion criteria: (1) non-smokers, (2) no current chronic diseases, (3) no current orthopedic injuries that prevent from maximal effort. The anthropometric and physiological characteristics of participants are presented in Table 1. All subjects gave written consent to participate in the study which was conducted in accordance with the local university’s ethical procedures and according to the Declaration of Helsinki.

### Procedures

Prior to data collection, measurements were taken from 32 sports facilities in the local area, to determine pitches size and their characteristics. The obtained information demonstrated that the pitches size ranged from 40 to 58 m in length and from 21 to 32 m in width, respectively, with length-to-width ratio ranging from 1.81 to 1.90. Pitches were usually sand carpet and artificial grass, and pitch perimeters were enclosed with wire netting just behind goals and side-lines.

Observations from these sports facilities proved that recreational participants mostly preferred 6-aside to 8-a-side games. The occupancy rate of the facilities was usually higher at night. Based on these considerations, the present study was carried out in two separate pitch sizes (one of the smallest and one of the largest) and with different numbers of players (5-a-side and 7-a-side, except for goalkeeper). Pitches size and their exact dimensions are shown in Table 2. The studied subjects took part in four separate games, each lasting 40 min, once a week in a random order. Except for goalkeepers, each team comprised 5 or 7 players who played on a small (S 5-a-side, S 7-a-side) and a large pitch (L 5-a-side, L 7-a-side). During 7-a-side games, only the players who played in 5-a-side games were considered. However, their playing positions were kept constant throughout all sessions. Two teams were formed that were similar in terms of age and physical characteristics. The technical and tactical abilities of the players were also taken into account subjectively, based on observations made during three games prior to the study. All the games were scheduled at the same time of the day (10:00 pm), and filmed by two fixed cameras. In addition, HR was recorded telemetrically throughout all games. The participants consumed the last meal at least 3 hours before the sessions, and refrained from intense physical exercise for 2 days prior to each session. The average temperature and relative humidity during the games were 23.3 ± 1.26 °C and 66.2 ± 3.86%, respectively (Hanna Instruments, HI 8564, Italy). A standardized warm-up consisting of 5 min jogging and 5 min dynamic stretching was performed before each match. To avoid any potential difference in the playing time, certain rules and issues were taken into account that ensured quick restart of the game after each stoppage: i) there was wire netting just behind each goal that prevented the ball travelling far from the pitch perimeter, and there were also spare balls behind each goal; ii) after each stoppage (free-kick, corners and each goal scored etc), the participants were encouraged to restart the game as quickly as possible; iii) there were no throw-ins (the players were allowed to hit the ball against the wire netting on both sides and then control it). Furthermore, taking into account mandatory stoppage conditions such as injury, the time of the cameras and telemetric system watches were synchronized in order to be able to establish corresponding HR data. Nevertheless, none of the games required such stoppages throughout the study.

### Anthropometric Measurements

Body height and body mass were measured using a calibrated electronic scale (Seca, France) to the nearest 0.1 cm and 0.1 kg, respectively, with the subject lightly dressed without shoes. Thickness of skinfolds (biceps, triceps, subscapular and suprailiac) was measured with a skinfold caliper (Holtain Ltd, UK) to the nearest of 0.2 mm, on the right side of the body, using a standard procedure. Skinfold thicknesses were measured in duplicate or triplicate, and the mean of the two closest values was used for the analysis. The sum of four skinfold thicknesses was used to calculate percentage of body fat with the use of the Durnin and Womersely (1974) equation.

### Determination of Resting HR and HRmax

Resting HR was recorded for 10 min in a supine position using a HR monitor (S610i, Polar Electro Oy, Kempele, Finland). The resting HR value corresponded to the minimal HR value observed during this period (Dellal et al., 2012). HRmax was determined using the maximal multistage 20 m shuttle run test (SRT) according to the 1 min protocol (Leger et al., 1998). For the SRT, a 20 m running course with 1 m turning area behind each of the end lines, marked by plastic tape and cones, was set up in the sports hall. Following an explanation of the SRT protocol, subjects ran back and forth between two end lines, exactly 20 m apart, in time with the audible signals. The frequency of the sound signals increased in such a way that running speed started at 8.5 km·h-1 and was increased by 0.5 km·h-1 each minute. The SRT was terminated when the subject could not maintain the pace of the sound signals for two consecutive shuttles, or else felt fatigue and stopped running voluntarily. Before the SRT, subjects were instructed to exert maximal effort. Subjects were also encouraged verbally throughout the SRT to maintain the required pace as long as possible and to produce maximal effort. During the SRT, HR was measured with a Polar HR monitor, and individual HRmax was determined as the highest HR recorded (Gavarry et al., 1998).

### Measurement and evaluation of HR and RPE responses to games

Following the warm-up, HR was recorded with a sampling frequency of 5 s using a Polar HR monitor (S610i, Polar Electro Oy, Kempele, Finland). Data were subsequently uploaded to a computer using a Polar infrared interface with the Polar precision performance software (Version 4.01.029, Polar Electro Oy, Kempele, Finland). The data were then exported to a Microsoft Excel worksheet, where the time spent within the low intensity zone (< 70%HRmax), moderate intensity zone (70–85% HRmax) and high intensity zone (> 85% HRmax) was determined. Reference HR values were calculated using the “Karvonen formula” by multiplying the HR reserve (HRmax - HRrest) by the factors 0.70 and 0.85, and adding these values to the HR at rest. The percentage of HR reserve (%HRres) was also calculated by the following formula: %HRres = (match mean HR - resting HR)/(HRmax - resting HR) × 100 (Karvonen et al., 1957). Data were quantified as mean HR and mean percentage of HRres. The percentage of time spent within each intensity zone was also calculated with respect to that achieved in the SRT protocol. RPE was assessed using the 15-point Borg scale (Borg, 1982). All participants were asked to state their mean RPE to match-play immediately upon completion of each match. The participants had been familiarized with the scale at the end of 3 games prior to the start of the study, and also at the end of their SRT protocol. To obtain reliable feedback from the players, they were instructed in how to use the scale properly prior to the familiarization sessions and the beginning of all matches according to standardized instructions for RPE (Borg, 1998).

### Recording matches and technical evaluations

All the games were filmed by two fixed cameras (Sony DCR-SR15E, Japan), positioned behind each goal, at an elevation of approximately 6 m. The video recordings were later replayed to evaluate the following technical actions using a hand notation system: a) ball possession, b) dribbling, c) successful pass, d) unsuccessful pass, e) tackle and f) shooting. Since these technical actions are frequently performed during games the assumption is that they are indicative of players’ enjoyment and satisfaction associated with games. Reliability assessment was performed by the re-analysis of a randomly designated match by the same observer, the analyses being performed one week apart. The number of exact agreements observed between each of the analysis sessions was used to determine the level of agreement for the evaluation of technical actions. To enable this calculation, an observation-by-observation breakdown of the results was obtained for each analysis. Then, the number of agreements observed between two analyses was calculated by using kappa statistic (Kelly and Drust, 2009). The reliability (kappa) values for technical actions ranged from 0.80 to 0.93. This is representative of an almost perfect strength of agreement according to Landis and Koch (1977).

### Statistical Analysis

Data are expressed as means ± standard deviation. The assumption of normality and homogeneity of variance were verified using the Kolmogorov-Smirnov and Hartley’s Fmax test, respectively. Two-way analysis of variance on repeated measures was performed to test the effects of pitch size and the number of players for all variables (within subject). Effect size (ŋ2) was also calculated for each significant difference. All statistical analysis was performed using SPSS for windows, version 15.0 (SPSS Inc., Chicago, USA). The level of statistical significance was set at p<0.05 and p<0.01.

## Results

Various HR and RPE responses for pitch size and the number of players are presented in Table 3. Analysis of variance revealed that cardiac responses were higher during 5-a-side compared to 7-a-side games [mean HR: p< 0.01, ŋ2 = 0.614; % HRres: p< 0.01, ŋ2 = 0.599; peak HR: p<0.05, ŋ2 = 0.473 and peak HR (%HRmax): p< 0.05, ŋ2 = 0.478]. As presented in Table 4, the results also showed that the players spent less time in the low-intensity (p<0.05, ŋ2 = 0.446) and more time in the high intensity (p< 0.05, ŋ2 = 0.387) zones during 5-a-side games compared to 7-a-side. However, time spent within the moderate-intensity zone was similar between games (p>0.05). HR responses when playing on a large pitch tended to be higher than for a small pitch, but the differences between the two pitches were non-significant (p>0.05). There were no significant differences in RPE values across all formats of games (p>0.05). The interaction between main effects was not significant for any of the HR variables and RPE (p>0.05).

The results of two-way ANOVA on technical actions are presented in Table 5.

The results indicated that the players performed more successful passes (p<0.01; ŋ2 = 0.659) and dribbling (p<0.05; ŋ2 = 0.478), and fewer unsuccessful passes (p<0.01; ŋ2 = 0.633) during 5-aside compared to 7-a-side games. Furthermore, ball possession (p<0.05; ŋ2 = 0.411) and unsuccessful passes (p<0.05; ŋ2 = 0.381) were higher on a small pitch compared to a large pitch. However, neither the number of players nor pitch size had significant effects on tackling and shooting (p>0.05). The interaction was not significant for any of the technical action examined (p>0.05).

## Discussion

The present study focused on the cardiac responses, perceived exertion and technical effects of altering pitch size and the number of players in recreational soccer match-play. The main findings suggest that the number of players is a primary factor affecting HR responses and technical actions during recreational soccer. The results also indicate that participation in all formats of recreational soccer should be expected to provide sufficient stimuli to improve cardiovascular fitness associated with high relative intensities of exercise.

HR responses during recreational soccer have been well documented (Bertolaccini et al., 2010; Bondarev, 2011; Randers et al., 2010; Randers et al., 2011; Boyd et al., 2012). Nevertheless, it is still unclear how cardiovascular responses are affected by altering pitch size and the number of players. The present findings demonstrated that addition of extra two players led to a decrease in cardiovascular responses, represented by average and peak HR, % HRres and time spent above 85% HRmax (Table 3). This finding is in line with previous studies of amateur and professional players, in which a decrease in the number of players resulted in elevated HR responses (Rampinini et al., 2007; Casamichana and Castellano, 2010; Owen et al., 2004). In this study, the higher number of ball possessions and dribbling during 5-a-side games would be expected to increase energy consumption (Reilly and Ball, 1984), thereby leading to increased cardiac response. Additionally, Jones and Drust (2007) stated that the requirement for high intensity effort increases when the number of players is reduced. However, in contrast to the current findings, a previous study reported no differences in HR response when comparing 7-a-side with 4-a-side and fewer players in untrained males (Randers et al., 2010). In addition, in a study conducted on physically active university students, Bondarev (2011) reported that the player’s effect had an influence on cardiac responses when the number of players was equal to or less than four players. In contrast to this conclusion, Rampinini et al. (2007) reported that both 4-a-side and 5-a-side games led to more marked physiological responses (HR, lactate and RPE) than 6-a-side games. On the other hand, the present results showed a non-significant increase in HR response for games played on a large pitch (Table 3). This finding is in line with previous studies conducted on recreational (Bondarev, 2011) and professional players (Kelly and Drust, 2009). However, a previous study of young soccer players found higher mean HR and time spent above 90% HRmax when the individual playing area was larger (Casamichana and Castellano, 2010). Rampinini et al. (2007) also reported that HR and blood lactate concentration were higher during small-sided games played on a large pitch than on medium-sized and small ones. Direct comparison between studies is difficult due to methodological variations, such as participants’ characteristics, the number of players, pitch size, game duration, rules and instructions. Consequently, the current findings suggest that, independent of pitch size, the cardiovascular demands imposed on participants increase when the game is played with fewer players.

In a recent literature review on soccer players, it was reported that both a decrease in the number of players and an increase in the playing area resulted in higher RPE responses (Aguiar et al., 2012). However, the present results indicated that, despite relatively high cardiac responses, RPE values were moderate and similar across all formats of games (Table 3). To our knowledge, only one study previously attempted to investigate RPE values with respect to the number of players in recreational games, and reported similar findings to the present study (Randers et al., 2010). In a study of young soccer players, Aslan et al. (2012) indicated that players perceived the periods of the game as being progressively more physically challenging, although they were less active. This implies that RPE might be used when assessing accumulated fatigue during intermittent prolonged exercise. However, in the current study, match duration (40 min) might be relatively short for the accumulation of fatigue.

This study also showed that the relative intensities of all formats of recreational match-play were higher than the minimum intensity level recommended by scientific institutions for cardiovascular fitness improvements (ACSM, 2000). This finding is in accordance with the findings of previous studies conducted on special groups with various training status, social background, sex, and age (Boyd et al., 2012, Randers et al., 2010; Bondarev, 2011; Bertolaccini et al., 2010). Thus, it can be suggested that all formats of recreational match-play can be used as an effective activity to promote cardiovascular fitness. However, as in this study, the majority of reports have demonstrated that certain periods of the games showed greater cardiovascular response than the intensity level recommended for physical fitness (Randers et al., 2010; Bondarev, 2011; Bertolaccini et al., 2010). It was reported that HR values of more than 85% of HRres potentially increase cardiovascular risk factors (ACSM, 2000). In a recent literature review, Le Goff et al. (2012) stated that intense physical exercise has an impact on cardiovascular function, represented by the plasma variations of some cardiovascular risk markers (cardiac troponin, myeloperoxidase, amino-terminal pro-brain natriuretic peptide, C-reactive protein and oxidized low-density lipoprotein). However, regularly performed moderate physical activity would be the main strategy for prevention of cardiovascular disease (ACSM, 2000). The current results showed that players spent 36.7% (14.6±5.8 min) and 38.4% (15.3±4.3 min) of total match time in the high-intensity zone (> 85% HRmax) during 5-a-side games on a small and large pitch, respectively. Corresponding values during 7-a-side games were 26.0% (10.4±3.3 min) and 31.7% (12.7±4.8 min), respectively (Table 4). In addition, during games, players showed peak HR similar to their HRmax and reached almost mean intensity of 80% HRres (Table 3) what is in accordance with previous findings (Randers et al., 2010; Bondarev, 2011; Bertolaccini et al., 2010). Similarly, a recent study clearly demonstrated that young players spent approximately 20% of total time with a HR above 90% of maximum. Corresponding values were 22%, 27%, 32 % and 48% for untrained man, homeless, middle age and elderly man, respectively (Randers et al., 2010). These values indicate that individual relative exercise intensity is considerably high during certain moments of small-sided games for diverse populations, especially among elderly. Moreover, even though standing steadily and very low intensity activities constitute an important proportion of total match time, mean HR exceeds 80% of HRmax for all levels of recreational players (Randers et al., 2010; Bondarev, 2011; Bertolaccini et al., 2010) and also among elite soccer players (Bangsbo, 1994). There are some possible reasons explaining this finding. First, besides running, players perform other game-related energy-demanding activities, such as backwards/sideways runs, dribbles, tackles, shots and turns that contribute to the overall demands on the players (Randers et al., 2010; Reilly and Ball, 1984). In addition, HR can be affected by several factors (increasing hormone concentration and hyperthermia, etc.) which can expand the mean values by causing the HR after high intensity activities to remain high even during subsequent low intensity activities (Aslan et al., 2012). Nonetheless, despite these intense periods and relatively high mean intensity, players’ RPE was at a moderate level during all formats of games (Table 3). A similar result was also observed in a study of male and female recreational players (Randers et al., 2010). This finding may imply that, even though relative physiological stress imposed on players was high, they could not accurately perceive their level of fatigue. Thus, depending on the motivational climate of the games, the players might overexert themselves. Such a situation may be potentially hazardous, and can cause undesirable cardiovascular events by diminishing players’ self-control. Therefore, participants should be aware of their limits to ensure the safety of an activity. This suggestion is especially relevant for participants who do not participate regularly in sport activity, or who are overweight and clinical (Boyd et al., 2012).

A few previous studies addressed the technical actions performed during various formats of recreational games (Randers et al., 2010). This may be because technical actions are not the major aim of recreational soccer. However, as mentioned earlier, individuals’ participation in an activity is not only related to a belief in health benefits but also for the enjoyment and satisfaction associated with it. The findings of this study demonstrated that, independent of pitch size, the players performed more successful passes and dribbling, and fewer unsuccessful passes during 5-a-side games compared to 7-a-side. Furthermore, technical actions were also influenced by pitch size in that the number of ball possessions and unsuccessful passes was higher on the small pitch. A study involving untrained males reported more tackles when playing 4-a-side or fewer players than for 7-a-side games (Randers et al., 2010). Jones and Drust (2007) reported that the number of individual ball contacts per game increased by reducing the number of players involved. A previous study of youth professional players also showed that additional players led to fewer technical actions performed per player (Owen et al., 2004). On the other hand, studies in soccer players indicated that increasing the size of the pitch had no significant effect on the technical actions performed (Kelly and Drust, 2009; Owen et al., 2004). Solely in terms of technical actions employed, the results of the present study may lead to the conclusion that players may have more chance to perform basic technical actions during 5-a-side games, especially on small pitches but also on large pitches. Thus, 5-a-side games in both pitch sizes could increase the enjoyment and satisfaction level of participants. Nonetheless, this issue requires more detailed analysis using larger research groups. In this study, technical actions were accepted as indicative of players’ enjoyment and satisfaction associated with match-play. However, it would also be of importance to evaluate players’ perceptions via physical activity enjoyment scales.

In conclusion, the present study suggests that the number of players is a primary factor affecting HR responses and technical actions during recreational soccer match-play. Independent of pitch size, the cardiovascular demands imposed on participants increase when the game is played with fewer players. Some technical actions also vary with the reduction of the number of players, with a significant increase in successful passes and dribbling, and with a significant decrease in unsuccessful passes. In addition, the number of ball possessions and unsuccessful passes increases as the pitch size decreases. However, these results are highly influenced by a number of factors, including age, gender, ability and the level of physical fitness. Hence, future studies should replicate these findings in diverse populations. The results also indicated that all formats of recreational soccer can be used as an effective activity to promote cardiovascular fitness associated with high relative intensities of exercise. However, during certain periods of all formats of games, relative exercise intensity exceeded the upper thresholds recommended for cardiovascular safety. The findings of the present study would suggest that relative cardiovascular demands (% HRres and time spend above 85% of HRmax) of games can be decreased slightly by increasing the number of players involved.

## Figures and Tables

**Table 1:** Characteristics of the participants (mean ± SD)

Age (years)	Body height (cm)	Body mass (kg)	Body Fat (%)	Maximum heart rate (beats · min^−1^)	Resting heart rate (beats · min^−1^)	Heart rate reserve (beats · min^−1^)
31.7±7.6	177.4±6.1	78.7±5.7	20.2±3.1	189.2±11.2	68.2±7.6	121.0±10.5

% Body Fat = calculated from the sum of four skinfold thicknesses.

Maximum heart rate = the highest heart rate recorded during shuttle-run test protocol.

Resting heart rate = the lowest heart rate recorded when the participants lay on supine position for 10 min.

Heart rate reserve = maximum heart rate – resting heart rate.

**Table 2:** The pitch sizes and their characteristics

Pitches	Pitch Location / Material	Pitch Size	Playing Area	Length / Width	Playing Area per player (5-a-side)	Playing Area per player (7-a-side)
Small Pitch	Outdoor / sand carpet	44 – 23	1012 m^2^	1.91	101.2 m^2^	72.3 m^2^
Large Pitch	Outdoor / sand carpet	57 – 30	1710 m^2^	1.90	171.0 m^2^	122.1 m^2^

**Table 3:** Mean and peak HR, and RPE during various format of recreational soccer. (mean ± sd)

Variables	S (5-a side)	S (7-a side)	L (5-a side)	L (7-a side)	F values		
					Player Effect	Pitch Effect	Player × Pitch Effect
Mean HR (b·min^−1^) ^a^	164.3±11.9	161.2±12.9	167.0±13.2	163.5±12.8	14.31	3.06	0.15
Mean HR (% HR_res_)^a^	79.4±3.7	76.8±4.4	81.7±4.7	78.7±4.3	13.42	3.02	0.16
Peak HR (b·min^−1^) ^a^	185.0±12.3	183.2±13.5	187.7±12.8	182.5±16.1	8.07	1.31	2.09
Peak HR (% HR_max_) ^a^	97.7±1.6	96.7±2.0	99.2±1.9	96.3±3.4	8.24	1.20	2.16
RPE	12.4±1.2	12.3±0.9	13.2±1.9	12.8±1.2	0.24	3.61	0.37

S: small pitch, L: large pitch, HR: heart rate, % HR_max_: percentage of maximum heart rate, RPE: rate of perceived exertion,

“a” denotes a significant player effect [p< 0.01 for mean HR and mean HR (%HR_res_) and p< 0.05 for peak HR and peak HR (%HR_max_)].

**Table 4:** Time spend at various percent of HR_max_ during recreational soccer. (mean ± sd)

Time Spend (% HR_max_)	S (5-a-side)		S (7-a-side)		L (5-a-side)		L (7-a-side)		F values		
	
	Min	%	Min	%	Min	%	Min	%	Player Effect	Pitch Effect	Player × Pitch Effect
< 70%^a^	6.2±2.7	15.3±6.9	7.7±2.7	19.1±6.8	5.4±2.7	13.4±6.8	8.6±3.3	21.6±8.3	7.25	0.04	0.59
70 – 85%	19.2±4.9	48.0±12	21.9±5.1	54.9±12.0	19.3±3.1	48.2±7.7	18.7±4.9	46.7±12.3	0.76	3.09	1.06
> 85%^a^	14.6±5.8	36.7±14	10.4±3.3	26.0±8.0	15.3±4.3	38.4±10.8	12.7±4.8	31.7±12.1	5.68	1.95	0.26

S: small pitch, L: large pitch, HR_max_: maximum heart rate,

“a” denotes a significant player effect at p< 0.05

**Table 5:** Frequency of technical actions performed during various formats of recreational soccer. (mean ± sd)

Technical Actions	S (5-a-side)	S (7-a-side)	L (5-a-side)	L (7-a-side)	F values		
					Player Effect	Pitch Effect	Player × Pitch Effect
Ball Possessions ^b^	47.4±10.8	45.0±7.1	43.4±11.1	40.5±11.5	2.46	6.27	0.01
Dribbling ^a^	13.9±7.9	12.1± 6.5	15.6±6.8	11.3±6.6	8.23	0.47	3.50
Successful pass ^a^	27.2±9.9	21.8±7.2	24.3±7.9	20.9±8.0	17.35	1.73	0.25
Unsuccessful pass ^a, b^	5.6±2.7	7.7±2.4	4.3±1.1	6.0±1.8	15.55	5.54	0.23
Tackle	5.1±2.6	6.2±2.1	6.2±4.3	5.8±2.6	0.33	0.12	0.73
Shot	6.5±3.9	4.8±2.5	4.9±2.7	4.1±2.4	1.90	3.26	0.77

S: small pitch, L: large pitch,

“a” denotes a significant player effect (p< 0.05 for dribbling and p<0.01 for successful and unsuccessful passes),

“b” denotes significant pitch effect (p< 0.05 for ball possessions and unsuccessful passes)

## References

[b1-jhk-38-95] ACSM (2000). ACSM’s Guidelines for exercise testing and prescription.

[b2-jhk-38-95] Aguiar M, Botelho G, Lago C, Maças V, Sampaio J (2012). A Review on the Effects of Soccer Small-Sided Games. J Hum Kinet.

[b3-jhk-38-95] Ali A, Farrally M (1991). Recording soccer players’ heart rates during matches. J Sports Sci.

[b4-jhk-38-95] Aslan A, Açıkada C, Güvenç A, Gören H, Hazır T, Özkara A (2012). Metabolic demands of match performance in young soccer players. J Sport Sci Med.

[b5-jhk-38-95] Bangsbo J (1994). The physiology of soccer: With special reference to intense intermittent exercise. Acta Physiol Scand.

[b6-jhk-38-95] Bertolaccini MS, Orsatti FL, Neto OB, Mendes EL, Penaforte FRO, Ide BN, Lopes CR, Mota GR (2010). Soccer only once a week generates excessive cardiac responses. J Health Sci Inst.

[b7-jhk-38-95] Bondarev DV (2011). Factors influencing cardiovascular responses during small-sided soccer games performed with recreational purposes. Physical Education of Students.

[b8-jhk-38-95] Borg G (1982). Psychophysical bases of perceived exertion. Med Sci Sports Exerc.

[b9-jhk-38-95] Borg G (1998). Borg’s perceived exertion and pain scales.

[b10-jhk-38-95] Boyd JC, MacMilan NJ, Green AE, Ross JED, Thorb DB, Gurd BJ (2012). Exercise intensity of recreational sport: Impacts of sex and fitness. J Sport Sci Med.

[b11-jhk-38-95] Casamichana D, Castellano J (2010). Time–motion, heart rate, perceptual and motor behaviour demands in small-sides soccer games: Effects of pitch size. J Sports Sci.

[b12-jhk-38-95] Coutts AJ, Rampinini E, Marcora SM, Castagna C, Impellizzeri FM (2009). Heart rate and blood lactate correlates of perceived exertion during small-sided soccer games. J Sci Med Sport.

[b13-jhk-38-95] Dellal A, Owen A, Wong DP, Krustrup P, Exsel MV, Mallo J (2012). Technical and physical demands of small vs. large sided games in relation to playing position in elite soccer. Hum Movement Sci.

[b14-jhk-38-95] Durnin JV, Womersley J (1974). Body fat assessed from total body density and its estimation from skinfold thickness measurements on 481 men and women aged 16 to 72 years. Br Med J.

[b15-jhk-38-95] Gavarry O, Bernard T, Giacomoni M, Seymat M, Euzet JP, Falgairette G (1998). Continuous heart rate monitoring over 1 week in teenagers aged 11±16 years. Eur J Appl Physiol.

[b16-jhk-38-95] Jones S, Drust B (2007). Physiological and Technical Demands of 4 v 4 and 8 v 8 games in elite youth soccer players. Kinesiology.

[b17-jhk-38-95] Karvonen MJ, Kentala E, Mutsala O (1957). The effects of training on heart rates. A “longitudinal” study. Annales Medicinae Experimentalis et Biologiae Fenniae.

[b18-jhk-38-95] Katis A, Kellis E (2009). Effects of small-sided games on physical conditioning and performance in young soccer players. J Sports Sci Med.

[b19-jhk-38-95] Kelly D, Drust B (2009). The effect of pitch dimensions on heart rate responses and technical demands of small-sided soccer games in elite players. J Sci Med Sport.

[b20-jhk-38-95] Krustrup P, Nielsen JJ, Krustrup BR, Christensen JF, Pedersen H, Randers MB, Aagaard P, Petersen AM, Nybo L, Bangsbo J (2009). Recreational soccer is an effective health promoting activity for untrained men. Br J Sports Med.

[b21-jhk-38-95] Landis JR, Koch GG (1977). The measurement of observer agreement for categorical data. Biometrics.

[b22-jhk-38-95] Leger LA, Mercier D, Gadoury C, Lambert J (1998). The multistage 20 metre shuttle run test for aerobic fitness. J Sports Sci.

[b23-jhk-38-95] Le Goff C, Laurent T, Kaux JF, Chapelle JP (2012). Intense physical exercise related to the emergent generation of cardio-vascular risk markers: A review. Biol.Sport.

[b24-jhk-38-95] Little T, Williams AG (2006). Suitability of soccer training drills for endurance training. J Strength Cond Res.

[b25-jhk-38-95] Martin DT, Andersen MB (2000). Heart rate-perceived exertion relationship during training and taper. J Sports Med Phys Fit.

[b26-jhk-38-95] Owen A, Twist C, Ford P (2004). Small-sided games: the physiological and technical effect of altering pitch size and player numbers. Insight.

[b27-jhk-38-95] Rampinini E, Impellizzeri FM, Castagna C, Abt G, Chamari K, Sassi A, Marcora SM (2007). Factors influencing physiological responses to small-sided soccer games. J Sports Sci.

[b28-jhk-38-95] Randers MB, Petersen J, Andersen LJ, Krustrup BR, Hornstrup T, Nielsen JJ, Nordentoft M, Krustrup P (2011). Short-term street soccer improves fitness and cardiovascular health status of homeless men. Eur J Appl Physiol.

[b29-jhk-38-95] Randers MB, Nybo L, Petersen J, Nielsen JJ, Christiansen L, Bendiksen M, Brito J, Bangsbo J, Krustrup P (2010). Activity profile and physiological response to football training for untrained males and females, elderly and youngsters: influence of the number of players. Scand J Med Sci Sports.

[b30-jhk-38-95] Reilly T, Ball D (1984). The net physiological cost of dribbling a soccer ball. Res Q Exercise Sport.

[b31-jhk-38-95] Toh SH, Guelfi KJ, Wong P, Fournier PA (2011). Energy expenditure and enjoyment of small-sided games in overweight boys. Hum Movement Sci.

[b32-jhk-38-95] Vallerand RJ, Salvy SJ, Mageau GA, Eliot AJ, Denis PL, Grouzet FME, Blanchard C (2007). On the Role of Passion in Performance. J Pers.

